# Role of an Aromatic–Aromatic Interaction in the Assembly and Trafficking of the Zebrafish Panx1a Membrane Channel

**DOI:** 10.3390/biom10020272

**Published:** 2020-02-11

**Authors:** Ksenia Timonina, Anna Kotova, Georg Zoidl

**Affiliations:** 1 Department of Biology, York University, Toronto, ON M3J 1P3, Canada; ktimonin@yorku.ca (K.T.); kotova@my.yorku.ca (A.K.); 2 Department of Psychology, York University, Toronto, ON M3J 1P3, Canada

**Keywords:** pannexin, integral membrane protein, structure–function, protein trafficking, N-glycosylation, co-localization, FRAP, FRET

## Abstract

Pannexin 1 (Panx1) is a ubiquitously expressed hexameric integral membrane protein known to function as an adenosine triphosphate (ATP) release channel. Panx1 proteins exist in unglycosylated core form (Gly0). They undergo critical post-translational modifications forming the high mannose glycosylation state (Gly1) in the endoplasmic reticulum (ER) and the complex glycosylation state (Gly2) in the Golgi apparatus. The regulation of transition from the ER to the cell membrane is not fully understood. Using site-specific mutagenesis, dye uptake assays, and interaction testing, we identified two conserved aromatic residues, Trp123 and Tyr205, in the transmembrane domains 2 and 3 of the zebrafish panx1a protein. Results suggest that both residues primarily govern the assembly of panx1a subunits into channels, with mutant proteins failing to interact. The results provide insight into a mechanism enabling regulation of Panx1 oligomerization, glycosylation, and trafficking.

## 1. Introduction

Pannexin 1 (Panx1) is an integral membrane glycoprotein forming a large pore membrane channel ubiquitously in the mammalian central nervous system (CNS) [1,2,3]. The protein shares sequence homology with the invertebrate gap junction proteins called innexins [4]. Panx1 is a hexameric voltage-gated and mechanosensitive channel involved in calcium and adenosine triphosphate (ATP) signaling, and it is sensitive to extracellular pH changes [5,6,7,8]. Due to glycosylation modifications, Panx1 exists in three forms: the unmodified nonglycosylated state (Gly0), the high mannose state (Gly1) formed in the endoplasmic reticulum (ER), and the complex glycosylated state (Gly2) formed in the Golgi apparatus [9,10,11]. The addition of large glycosylation complexes is discussed as a reason preventing Panx1 from forming gap junctions. These modifications enable Panx1 to exchange small molecules between extracellular and intracellular environments [12]. A glycosylation-deficient mutant of mouse Panx1^N254Q^ demonstrated limited membrane localization, with only a subpopulation reaching the cell surface [10,11]. Despite Panx1’s limited capacity to form gap junctions, they are topologically similar to the gap junction protein family of connexins and innexins [4,5,13]. A single Panx1 subunit has four transmembrane domains, two extracellular loops, a cytoplasmic loop, and intracellular amino and carboxyl terminals. The exact structure and folding of the protein remain unknown as the structure of Panx1 at an atomic resolution has not yet been established.

Previously, a zebrafish (*Danio rerio)* Panx1 ortholog (panx1a) has been described and found to form functional membrane channels in the Neuroblastoma 2a (Neuro 2a) cell line [14,15]. Due to a teleost whole-genome duplication event that occurred between 320 and 350 million years ago, a panx1a ohnologue exists (panx1b) [16]. Panx1a and panx1b show distinct tissue expressions, glycosylation patterns, and electrophysiological gating properties [14]. This study will focus on the panx1a ohnologue.

Electrophysiological gating properties and multiple factors regulating the Panx1 channel, as well as complex pharmacology, have been described [17,18]. Panx1 blockers include carbenoxolone, mefloquine, and flufenamic acid, which also act on gap junction proteins. Potassium and glutamate can activate pannexins. Studies across multiple fields demonstrated that Panx1 is a major molecular hub interacting with many signaling pathways. To address the complex life cycle of Panx1, we explored the role of an aromatic–aromatic interaction between amino acids W123 and Y205 in the cytoplasmic loop of panx1a near transmembrane (TM) domains 2 and 3, respectively. Aromatic–aromatic interactions have been previously shown to be important in TM–TM association of membrane proteins [19,20]. The forces of these interactions help strengthen oligomerization and suggest a role in folding and stabilization. Both W123 and Y205 are highly conserved between various membrane channels and gap junction proteins. Mutation analysis of panx1a paired with co-localization and protein interaction studies led to the conclusion that the two aromatic residues are vital for the structural stabilization and interaction of panx1a TM domains before insertion into the cell membrane. Outcomes of this study are relevant to understand how Panx1 proteins mature and traffic to the cell membrane.

## 2. Materials and Methods

### 2.1. Plasmid Construction and Mutagenesis

The full-length *Danio rerio panx1a* wild type (WT) open reading frame (amino acids 1-416) was cloned into the enhanced yellow fluorescent protein plasmid (pEFYP-N1) expression vector (Clontech Laboratories Inc., Mountain View, CA, USA) as described [4]. For Förster Resonance Energy Transfer (FRET) analysis, the same sequence was cloned into pDsRed-monomer-N1 (Clontech Laboratories Inc., Mountain View, CA, USA). For protein interaction studies, *panx1a* WT and mutants were cloned into a pdTomato-His expression vector. For localization studies, ER and Golgi organelle markers tagged with DsRed2 were generated as described [21]. Mutagenesis was performed using the Q5 Hot Start Site-Directed Mutagenesis kit (New England Biolabs Inc., Boston, MA, USA) according to the manufacturer’s protocol. Oligonucleotides (Table 1) were designed using NEBaseChanger tool and synthesized by Integrated DNA Technologies (IDT, Coralville, IA, USA). All mutations were confirmed by double-stranded DNA sequencing (Eurofins, MWG Operon LLC, Huntsville, AL, USA).

### 2.2. Cell Culture and Transfection

Neuroblastoma 2a (Neuro 2a) cells were cultured in DMEM containing 2mM glutamine, 1% nonessential amino acids, 1% penicillin and streptomycin, and 10% fetal bovine serum. Cells were maintained at 37 °C in a humidified atmosphere with 5% CO_2_. For Western blots, ~50,000 cells were seeded in 24-well plates. For imaging of fixed cells and live-cell imaging experiments, ~25,000 cells were seeded in 24-well plates or 35mm glass-bottom dishes (MatTek Corporation, Ashland, MA, USA). For Ni-NTA His pull-down assays, a combined total of ~3,000,000 cells were seeded in two 100mm plates. For the cell surface biotinylation assay, ~800,000 cells were seeded in 60mm plates.

Neuro 2a cells were transiently transfected using the Effectene™ Transfection Reagent (Qiagen Inc., Valencia, CA, USA) according to the manufacturer’s protocol. For 24-well plates and 35mm glass-bottom dishes, cells were transfected with 200ng DNA for single transfections and 400ng DNA for double transfections. For each 100mm plate, 2000ng of DNA was used for single transfections, and 4000ng DNA was used for double transfections. For 60mm plates, 600ng of DNA was used. Expression and functional studies were performed 48 h post-transfection. For live microscopy experiments, the medium was changed for DMEM lacking phenyl red at least 30min prior to imaging.

### 2.3. Western Blot

Proteins were separated with 10% sodium dodecyl sulfate polyacrylamide gel electrophoresis (SDS-PAGE) at 100-150V for 1.5 h. The gel was placed onto a nitrocellulose membrane, and proteins were transferred using the Trans-Blot Turbo Transfer (Bio-Rad Inc., Mississauga, ON, Canada) system. Transfer conditions were 1.3A and 2.5V for 7 min. The membrane was blocked with Odyssey blocking buffer (LI-COR Biosciences, St. Lincoln, NE, USA) for 1h and incubated overnight with specific antibody mixtures. Primary antibodies used included rabbit anti-green fluorescent protein (GFP) (1:500) (Santa Cruise Biotechnologies, Dallas, TX, USA), mouse anti-GFP (1:500) (Roche Diagnostics Deutschland GmbH, Mannheim, Germany), rabbit anti-His (1:1000) (Bethyl Laboratories Inc., Montgomery, TX, USA), and mouse anti-beta-actin (1:15000) (Sigma-Aldrich Chemie GmbH, Munich, Germany). Secondary antibodies were donkey anti-rabbit IRDye680LT (1:15000) (LI-COR Biosciences) and goat anti-mouse IRDye800CW (1:15000) (LI-COR Biosciences). Membranes were imaged using an Odyssey^®^ CLx Infrared Imaging System (LI-COR Biosciences).

### 2.4. Immunofluorescence, Confocal Microscopy, and Co-localization

For immunofluorescence, transfected cells were fixed with 100% methanol for 10 min at room temperature (RT). Cells were blocked in Phosphate-buffered saline (PBS) containing 2% bovine serum albumin (BSA) for 1h at RT. Next, the primary antibody dilution containing rabbit anti-His (1:1000) (Bethyl Laboratories Inc.) in PBS with 0.1% BSA was applied for 1h at RT. Cells were then incubated with 2ug/mL of secondary antibody dilution containing Alexa Fluor 568 goat anti-mouse (Thermo Fisher Scientific, Rockford, IL, USA) in PBS with 0.1% BSA for 1h at RT. Lastly, cells were washed in PBS and mounted with Fluoroshield^TM^ (Sigma-Aldrich).

For imaging of fluorescently tagged proteins, transfected cells were fixed with 4% paraformaldehyde for 30 min at RT and mounted with Fluoroshield^TM^ (Sigma-Aldrich). Images were taken with a Zeiss LSM 700 confocal microscope using a Plan-Apochromat 63x/1.4 Oil DIC M27 objective. Zeiss ZEN imaging software was used to control all imaging parameters.

### 2.5. His60 Ni Gravity Column Pull Down

Neuro 2a cells were double transfected with either WT panx1a-dTomato-His and WT panx1a-EYFP, Y205A-dTomato-His and WT panx1a-EYFP, or dTomato-His with WT panx1a-EYFP. The cells were lysed under native conditions using His60 Ni xTractor^TM^ Buffer provided with the His60 Ni Gravity Column Purification kit according to the manufacturer’s protocol (Clontech Laboratories Inc.). The lysates were loaded onto equilibrated columns and rocked at 4 °C for 1h. The columns were washed, and proteins were eluted according to the manufacturer’s protocol. All samples were recovered in 1X Laemmli sample buffer, heated at 95 °C for 5–10 min, and analyzed by Western blotting.

### 2.6. Cell Surface Biotinylation Assay

Neuro 2a cells transfected with fluorescently tagged WT panx1a or mutants were subject to cell surface biotinylation assay 48 h post-transfection. Cells were grown on 60mm plates and washed once with warm PBS containing calcium and magnesium. Cells were then labeled with 0.25mg of membrane-impermeable EZ-link^TM^ Sulfo-NHS-Biotin (Thermo Scientific) per plate for 30 min at room temperature. The reaction was quenched with three washes of 50mM glycine buffer for a total of 15 min at RT. Cells were collected in PBS lacking calcium and magnesium, washed, and lysed in NP40 lysis buffer for 30 min on ice with periodic vortexing. Cells were then incubated with 90uL of prewashed Dynabeads^TM^ MyONE^TM^ Streptavidin C1 (Invitrogen) on a shaker at 4 °C overnight. Bead complexes were washed with the following buffers: buffer 1 (2% SDS in dH20), buffer 2 (0.1% deoxycholate, 1% Triton X-100, 500mM NaCl, 1mM EDTA, 50mM Hepes; pH 7.5), buffer 3 (250mM LiCl, 0.5% NP-40, 0.5% deoxycholate, 1mM EDTA, 10mM Tris; pH 8.1), and buffer 4 (50mM Tris, 50mM NaCl pH 7.4). Bead complexes were then resuspended in 60uL of 1X Laemmli buffer and boiled for 5 min to elute the proteins. Eluted proteins were analyzed by Western blot analysis using rabbit anti-GFP (1:500) (Santa Cruise Biotechnologies) to detect wild type and mutant panx1a-EYFP. A mouse anti-beta-actin antibody (1:15000) (Sigma-Aldrich) was used to as reference protein.

### 2.7. Pharmacology

Transfected cells were treated with 5ug/mL Brefeldin A (BFA) (Sigma-Aldrich) for 19h before localization studies and Western blot analysis. BFA has been used as an inhibitor for the transport of Panx1 between the ER and Golgi compartments [9].

### 2.8. Dye Uptake Assay

Neuro 2a cells transfected with fluorescently tagged WT panx1a and panx1a mutants were tested for dye uptake 48 h post-transfection using a protocol described previously [14,22]. Cells were grown on 35mm glass-bottom dishes and were incubated in clear DMEM lacking phenol red for 30min prior to imaging. Dishes were placed in a live-cell imaging chamber at 37 °C with CO_2_ using a Zeiss 700 confocal microscope. Ethidium bromide (EtBr) was added to a final concentration of 10uM immediately prior to imaging. Images were taken every 1 min for a duration of 10 min at a 512 × 512 pixel resolution with a scan speed of 4. Fluorescent values after 10 min of EtBr uptake were subject to statistical analysis.

### 2.9. Fluorescence Recovery After Photobleaching (FRAP)

Neuro 2a cells were grown on 35mm glass-bottom dishes and transfected with fluorescently tagged WT or mutant panx1a to assess cell surface dynamics [21]. Cells were incubated in DMEM lacking phenol red for 30 min prior to experimentation. Dishes were placed into a 37 °C live-cell imaging chamber, and time-lapse imaging was performed using a Zeiss 700 confocal microscope. Regions of interest (ROIs) were selected at the membrane of single cells with no neighboring cells. ROIs were photobleached at 488nm at 100% laser strength. Images were taken every 1 s for 50 s post bleaching. Recovery was calculated using the following equation:

*F* = (*Ft* − *F*0)/(*Fi* − *F*0) (1)

where F is the normalized fluorescence at a given time point, *Ft* is the fluorescence intensity at *t* seconds, *F0* is the fluorescence intensity upon bleaching, and *Fi* is the fluorescence intensity immediately prior to bleaching.

### 2.10. Förster Resonance Energy Transfer (FRET)

Neuro 2a cells were transfected with combinations of EYFP and DsRed-tagged WT and mutant panx1a using a previously established protocol [21]. Cells were fixed on coverslips, and mounted slides were placed in the Zeiss LSM 700 confocal microscope. Baseline readings were measured prior to the acceptor bleach protocol. The 555nm laser was set to 100% to photobleach the DsRed-tagged proteins until a 90% reduction of initial intensity was reached. The resulting intensity of the EYFP-tagged proteins was then measured using the 488nm laser. FRET efficiency was calculated using the FRET efficiency formula:

*FRETeff* = (*Dpost* − *Dpre*)/*Dpost* (2)

where *Dpost* is the average intensity after the bleach, and *Dpre* is the average intensity before the bleach. The threshold value of 10 nm distance was converted into FRET efficiency and was calculated to be 1.4% for DsRed and EYFP pair, based on the reference distance between the two fluorescent tags (4.9 nm) [23]. FRET distance was calculated using the formula:

*R* = *R_o_*((1/*E*) – 1)^1/6^ (3)

where *R_o_* is the distance between two fluorescent tags, *E* is the FRET efficiency, and *R* is the FRET distance.

### 2.11. Quantitative Real-Time PCR

Total RNA was extracted 48h post-transfection using RNeasy Plus Mini Kit (Qiagen) according to the manufacturer’s protocol from Neuro 2a cells, with or without 5ug/mL BFA treatment for 19h. A total of 1ug of RNA was used to synthesize cDNA using the ReadyScript cDNA Synthesis Kit (Sigma-Aldrich). qPCR was performed using the SsoFast EvaGreen Supermix (Bio-Rad) with the oligonucleotide pairs described (Table 2). Quantification of 18s rRNA served as an internal standard. Each assay was performed in triplicate in three independent experiments using the CFX Connect Real-Time PCR Detection System (Bio-Rad). Relative gene expression values were calculated using the Relative Expression Software Tool (REST) [24] with the EYFP-transfected cells serving as the control group.

### 2.12. Statistical Analysis

Statistical analysis was performed using MatLab R2018b, GraphPad Prism 8, or the Relative Expression Software Tool (REST) [24]. Data were analyzed for statistical significance using the Wilcoxon–Mann–Whitney test. All graphs were generated using GraphPad Prism. Co-localization data were generated using ImageJ software. Quantitative real-time PCR and co-localization results were expressed as mean ± standard error of the mean (SEM). All other results are expressed as mean ± standard deviation (SD). All data were generated from at least three independent experiments.

## 3. Results

### 3.1. Mutation of Aromatic Amino Acids Alters Trafficking of Panx1a to the Cell Membrane

**Figure 1:**
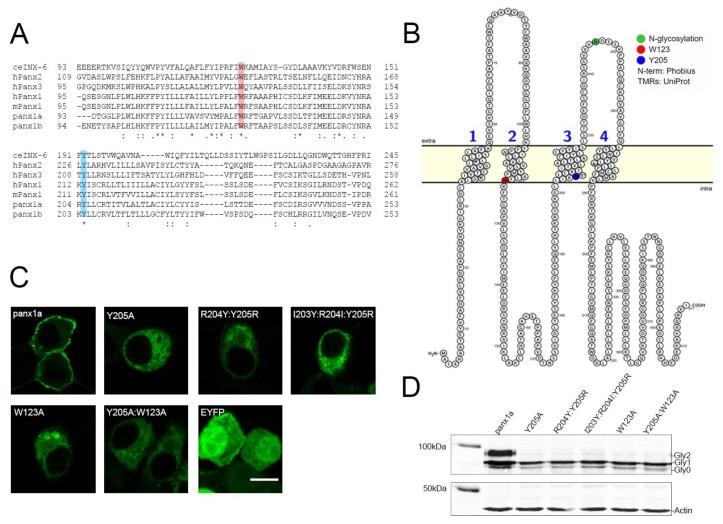
Expression and localization of panx1a WT and mutants in Neuro 2a cells. (**A**) Sequence alignment of panx1a with pannexin orthologs and paralogs and the *Caenorhabditis elegans* INX-6 protein. (**B**) Topological representation of a panx1a subunit showing the positions of both aromatic residues at the transmembrane and intracellular loop borders. The image was generated using Protter [27]. (**C**) Localization of EYFP-tagged wild type panx1a and mutants in transfected Neuro 2a cells. Scale bar: 10µm. (**D**) Western blot analysis of transfected EYFP-tagged panx1a and mutants. The three bands correspond to nonglycosylated panx1a (Gly0), the high mannose glycosylation (Gly1), and the complex glycosylation (Gly2) forms. Expressed proteins were detected using an anti-GFP antibody and an anti-ß-actin antibody as the control.

Sequence alignment of multiple pannexin orthologs and paralogs, as well as the distant invertebrate relative INX-6, revealed that aromatic amino acids W123 and Y205 of panx1a were conserved (Figure 1A). W123 and Y205 were located at the border of the predicted cytoplasmic loop and transmembrane domains 2 and 3, respectively (Figure 1B). The two amino acid residues aligned with the invertebrate INX-6 Y192 and W126. Previous work of Oshima et al. (2016) [25,26] showed that the Y192 of INX-6 was in close proximity to W126, leading us to propose that this also applies to the aromatic residues in panx1a. A topological comparison of orthologs and paralogs of panx1a and the distant invertebrate ortholog INX-6 showed that aromatic residues tyrosine and tryptophan appeared in similar positions (Supplementary Figure S1).

In Neuro 2A cells, the membrane localization of EYFP-tagged panx1a was compromised when mutating either Y205 or W123, thus identifying them as amino acids required for cell surface localization (Figure 1C). Substitution of these amino acids with an alanine residue resulted in disruption of membrane localization of panx1a, showing retained cytoplasmic expression. A positional shift of Y205 either one or two amino acids upstream of its original position resulted in a similar phenotype as the alanine substitution. These mutants were denoted R204Y:Y205R and I203Y:R204I:Y205R. A double alanine mutant at both Y205 and W123, denoted Y205A:W123A, also revealed cytoplasmic expression. The subcellular localization of mutants indicated that the position for this aromatic amino acid was critical.

The efficient membrane trafficking of panx1a requires N-glycosylation. A Western blot analysis determined the state of glycosylation for the aromatic mutants. The Y205A, R204Y:Y205R, I203Y:R204I:Y205R, W123A, and Y205A:W123A mutants showed a loss of the complex glycosylation (Gly2) form of the protein. All mutants still expressed the high mannose glycosylation form (Gly1) (Figure 1D).

### 3.2. Y205 Phosphorylation Is Not Required for Membrane Expression

**Figure 2:**
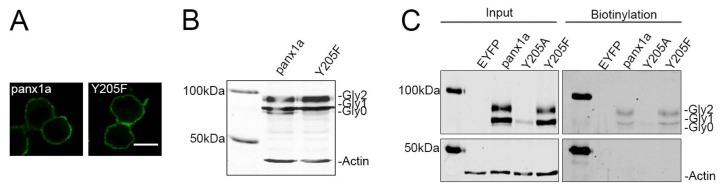
Comparison of the panx1a Y205F and Y205A mutants. (**A**) Neuro 2a cells transfected with WT panx1a-EYFP (left) and the Y205F-EYFP mutant (right) both showed membrane localization. Scale bar: 10µm. (**B**) Western blot analysis revealed similar glycosylation patterns when the Y205F mutant was compared to WT panx1a. (**C**) Cell surface biotinylation assay showing that Y205F was present at the membrane of transfected Neuro 2a cells. Cell lysates after NP40 buffer lysis (Input) showed expression of both intracellular (ß-actin; Y205A) and membrane proteins (panx1a; Y205F). The streptavidin pull-down fractions (Biotinylation) showed that all intracellular proteins were depleted, with panx1a and Y205F found in the protein fraction from the cell surface. ß-actin served as an internal control, showing no bands in the biotinylation fraction. Expressed proteins were detected using an anti-GFP antibody and an anti-ß-actin antibody as the control.

The possibility of Y205 being a site for tyrosine phosphorylation in panx1a was tested by replacement with the aromatic amino acid phenylalanine, which cannot be phosphorylated. Unlike Y205A, the EYFP-tagged Y205F mutant localization was similar to wild type panx1a (Figure 2A). Western blot analysis also showed that the Y205F mutant was efficiently glycosylated (Figure 2B), whereas Y205A was not. This was shown by the presence of the complex glycosylation variant of the panx1a protein. It was concluded that the presence of aromatic amino acids, but not phosphorylation of Y205, was required for membrane localization. The localization was confirmed by a cell surface biotinylation assay, where the Y205F mutant was biotinylated at the cell surface and pulled down with streptavidin (Figure 2C). Cells transfected with EYFP alone were used as a control, showing that the bands were specific to panx1a-EYFP. ß-actin, serving as endogenous quality control, was not detected in the biotinylated samples, indicating that biotin had not penetrated the membrane.

### 3.3. The Y205F Mutation Restores Panx1a Channel Function and Cell Surface Transport

**Figure 3:**
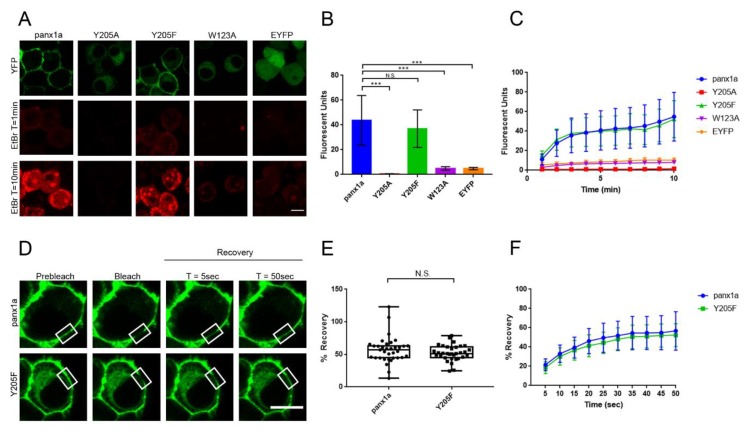
Functional analysis of the Y205F mutant using dye uptake assay and Fluorescence Recovery After Photobleaching (FRAP). (**A**) Fluorescent images are showing dye uptake of panx1a WT and mutants 10 min after EtBr application. EYFP-transfected cells were used as a negative control. Scale bar: 10µm. (**B**) Total EtBr fluorescence measured 10 min post dye application shown as a bar graph. Wild type panx1a: *n* = 17, Y205A: *n* = 14, Y205F: *n* = 16, W123A: *n* = 13, EYFP: *n* = 13. (**C**) EtBr fluorescence measured throughout the 10 min with one-minute intervals. (**D**) FRAP analysis of WT panx1a and Y205F mutant showing selected regions (white rectangles) pre bleaching, immediately after bleaching, and recovery 5 s and 50 s post bleaching. Scale bar: 10um. (**E**) Total % recovery, 50 s post photobleaching. Wild type panx1a: *n* = 35, Y205F: *n* = 33. (F). % recovery measured throughout 50 s with five-second intervals. Error bars show the standard deviation of the mean. ***p < 0.001, N.S., not significant.

A dye uptake assay and Fluorescent Recovery After Photobleaching (FRAP) analysis were used to assess the channel function and the cell surface dynamics of panx1a and mutants. As expected, both Y205A and W123A mutants were unable to uptake EtBr due to the lack of transport to the cell membrane. Fluorescent images showed the dye uptake at 0 (start) and 10 min (endpoint) post-treatment with EtBr (Figure 3A). Only the wild type and Y205F mutant showed dye uptake above baseline. When dye uptake was quantified, a significant difference in total EtBr uptake between WT (43.67 ± 20.00, *n* = 17) and both Y205A and W123A mutants was detected (Y205A: 0.60 ± 0.19, *n* = 14, *p* = < 0.0001; W123A: 4.99 ± 1.40, *n* = 13, *p* = < 0.0001) (Figure 3B). Both mutants showed similar dye uptake to the EYFP control (EYFP: 4.79 ± 0.97, *n* = 13, *p* = < 0.0001), which was considered to represent baseline uptake of EtBr. The Y205F mutant showed no significant difference in total dye uptake when compared to wild type panx1a (Y205F: 36.99 ± 15.13, *n* = 16, *p* = 0.38). The general trend of the dye uptake over the course of 10 min was similar for wild type and the Y205F mutant (Figure 3C).

Next, we assessed the cell surface diffusion dynamics of Y205F and wild type panx1a using FRAP. Images showed EYFP tagged wild type panx1a and Y205F expressing cells prior to bleaching, at bleaching, and recovery 5 s and 50 s post bleaching (Figure 3D). The average percent recovery 50 s post bleaching showed no significant difference between wild type panx1a and Y205F (WT: 56.70 ± 20.04, *n* = 35; Y205F: 52.28 ± 11.96, *n* = 33; *p* = 0.43) (Figure 3E). The general trend in recovery at a five-second interval was the same for both wild type and mutant proteins (Figure 3F). These results proved that the aromatic amino acid tyrosine could be substituted for phenylalanine at position 205 without disruption of channel mobility. Together with the dye uptake assay, these results showed that the Y205F mutant demonstrates wild type properties.

### 3.4. Y205A Is Retained in Intracellular Compartments but Does Not Induce ER Stress

**Figure 4:**
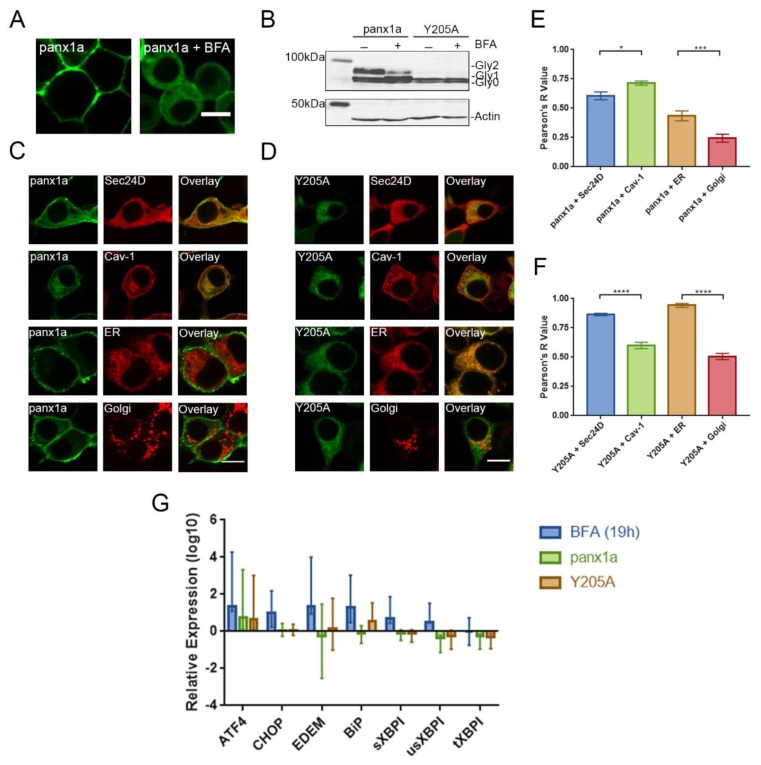
Expression of Brefeldin A (BFA)-treated cells, co-localization with cellular markers, and ER stress analysis of panx1a and the Y205A mutant. (**A**) Images of panx1a-EYFP transfected Neuro 2a cells treated with 5ug/mL BFA for 19h showed a reduction of cell membrane localization. Scale bar: 10µm. (**B**) Western blot analysis showed that treatment with 5ug/mL BFA for 19h caused a decrease in the Gly2 state and an increase in the Gly1 state of the wild type panx1a protein. No effect was detected for the Y205A mutant. Proteins were detected using an anti-GFP antibody. ß-actin served as a protein loading control. (**C**, **D**) Co-localization of EYFP-tagged panx1a and the Y205A mutant with mCherry-tagged Sec24D (COP II vesicle marker), His-tagged Cav-1 (Caveolin-1 marker), DsRed-tagged calreticulin (ER marker), and DsRed-tagged galactosyltransferases (Golgi apparatus marker). His-tagged Cav-1 was detected using an anti-His antibody and Alexa Fluor 568 secondary antibody. (**E**, **F**) Co-localization quantification of the wild type and mutant panx1a with the vesicle and organelle markers. Error bars show standard error of the mean. WT panx1a + Sec24D: *n* = 34, WT panx1a + Cav-1: *n* = 36, WT panx1a + ER: *n* = 29, WT panx1a + Golgi: *n* = 35, Y205A + Sec24D: *n* = 30, Y205A + Cav-1: *n* = 31, Y205A + ER: *n* = 20, Y205A + Golgi: *n* = 20. (**G**) Real-time qPCR analysis of ER stress genes. Neuro 2a cells were transfected with EYFP alone, panx1a-EYFP, and Y205A-EYFP. When indicated, cells were treated with 5ug/mL BFA for 19h. 18s rRNA was used as the reference gene. EYFP-transfected cells were used as the control group. Data were collected in three independent experiments in triplicate for each gene. All data were relative to the EYFP control group. ****p < 0.0001, ***p < 0.001, *p < 0.05.

First, we confirmed that the complex glycosylation of zebrafish panx1a was added in the Golgi apparatus by treating panx1a-EYFP transfected Neuro 2a cells with Brefeldin A (BFA). Previous studies have shown that 21 h treatment of 5ug/mL BFA disrupts the trafficking of rodent pannexin-1 between the ER and Golgi [9]. This was evidenced by Western blot showing a partial or complete decrease of the Gly2 form of the protein and a slight increase of the Gly1 form. Here, a 19h treatment with 5ug/mL BFA established a panx1a localization similar to the one reported for the mammalian pannexin-1. (Figure 4A,B). The localization of the Y205A-EYFP mutant was not affected by BFA treatment.

To test whether Y205A is retained in the intracellular compartments, co-localization studies with organelle and vesicle biomarkers were performed. Neuro 2a cells were double transfected with panx1a-EYFP WT and either Sec24D-mCherry (COP II marker), Cav-1-His (Caveolin-1 marker), calreticulin-DsRed (ER marker), or galactosyltransferase-DsRed (Golgi apparatus marker) (Figure 4C). The same was done with the Y205A-EYFP mutant (Figure 4D). Panx1a co-localized significantly with Cav-1, which was evident by the significant localization in the cell membrane. The Y205A mutant was visually co-localized most with the ER and Sec24D markers and less with the Golgi and Cav-1 markers. The quantification of co-localization showed panx1a co-localized significantly more with Cav-1 than Sec24D (Sec-24D: 0.61 ± 0.20, *n* = 34; Cav-1: 0.71 ± 0.12, *n* = 36; *p* = 0.018), and with ER than Golgi biomarkers (ER: 0.43 ± 0.23, *n* = 29; Golgi: 0.24 ± 0.19, *n* = 35; *p* = 0.0003) (Figure 4E). In contrast, the Y205A mutant co-localized significantly more with Sec24D than Cav-1 (Sec24D: 0.86 ± 0.055, *n* = 30; Cav-1: 0.60 ± 0.15, *n* = 31; *p* < 0.0001), and with ER than Golgi markers (ER: 0.94 ± 0.07, *n* = 20; Golgi: 0.51 ± 0.12, *n* = 20; *p* < 0.0001), suggesting that trafficking of Y205A out of the ER was impaired (Figure 4F).

Next, ER stress was ruled out by quantitative real-time PCR of established biomarkers (Figure 4E). The mRNA levels of five different ER stress markers were quantified: ATF-4, CHOP, EDEM, BiP, and XBP1 (spliced (s), unspliced (us), and total(t)). We used Neuro 2a cells treated with 5ug/mL BFA for 19h as the “stress” group and EYFP-transfected cells as the control group. The BFA-treated “stress” cells showed significant upregulation of all stress genes tested compared to the EYFP-transfected cells (ATF-4: *p* < 0.001, CHOP: *p* < 0.000, EDEM: *p* < 0.000, BiP: *p* < 0.000, sXBPI: *p* < 0.000, usXBPI: *p* = 0.019). Except for one upregulated gene, expression of Y205A did not affect cell stress markers (BiP: *p* = 0.005). Overexpression of both wild type and mutant panx1a caused no significant difference in the expression levels of stress genes when compared to EYFP-expressing cells.

### 3.5. Trafficking of Y205A to the Cell Surface Cannot Be Rescued by WT Panx1a

**Figure 5:**
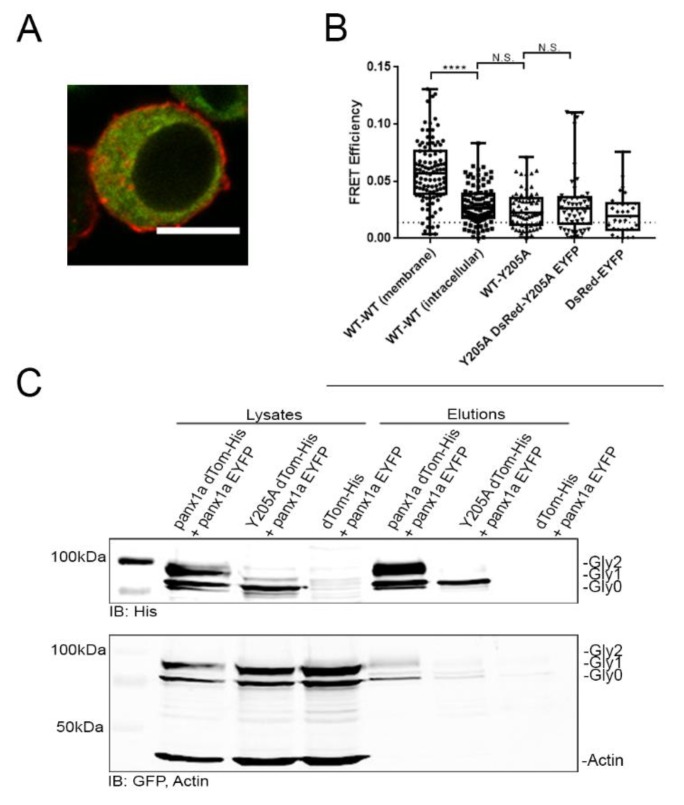
Interaction studies of panx1a and the Y205A mutant using FRET and pull-down assay. (**A**) Fluorescent image showing a Neuro 2a cell transfected with panx1a-DsRed and Y205A-EYFP. Scale bar: 10µm. (**B**) FRET efficiencies. WT–WT (membrane) (*n* = 103) indicates panx1a-DsRed interaction with panx1a-EYFP using selected cell membrane regions as a positive control. WT–WT (intracellular) (*n* = 100) indicates panx1a-DsRed interaction with panx1a-EYFP using selected regions inside the cell. WT-Y205A (*n* = 63) indicates panx1a-DsRed interaction with Y205A-EYFP inside the cell. Y205A-Y205A (*n* = 50) indicates Y205A-DsRed interaction with Y205A-EYFP inside the cell. DsRed-EYFP indicates DsRed interaction with EYFP inside the cell as the negative control. The dotted line represents the FRET efficiency threshold of 1.4%. (**C**) His60 Ni pull-down using cells double-transfected with panx1a-dTomato-His and panx1a-EYFP, Y205A-dTomato-His and panx1a-EYFP, or dTomato-His with panx1a-EYFP. Cell lysates on the left show expression levels before pull down. Elutions on the right show proteins recovered by pull down. Anti-GFP and anti-His antibodies detected eluted proteins. An anti-ß-actin antibody served as a loading control. ****p < 0.0001, N.S., not significant.

Double transfection of WT panx1a-DsRed with Y205A-EYFP did not rescue the observed localization phenotype. The intracellular retention of the mutant remained (Figure 5A). However, with the evident co-localization of mutant and WT proteins in the cytoplasm, we proceeded to test whether the two proteins interacted.

Förster Resonance Energy Transfer (FRET) experiments determined the proximity between panx1a WT and the Y205A mutant. Panx1a-DsRed and panx1a-EYFP had a FRET efficiency above the threshold of 0.014 at the cell membrane. We concluded that the channels had assembled in a compact state after insertion into the cell membrane (0.06 ± 0.03, *n* = 103) (Figure 5B). As expected, the intracellular FRET efficiency between panx1a-DsRed and panx1a-EYFP was significantly lower than at the cell membrane. This was explained as reflecting the increased compactness when panx1a channels were assembled from protein monomers (0.03 ± 0.02, *n* = 100; *p* = < 0.0001). No significant difference in FRET efficiency was detected between the intracellular panx1a-DsRed and panx1a-EYFP pair when compared to panx1a-DsRed paired with Y205A-EYFP mutant (0.03 ± 0.02, *n* = 63; *p* = 0.25), or pairs of mutants Y205A-DsRed and Y205A-EYFP (0.03 ± 0.03, *n* = 50; *p* = 0.71), suggesting neither combination had the ability to reach a compact state. To complement these results, we also calculated the FRET distance based on the FRET efficiencies (Supplementary Figure S2).

The FRET experiment did not allow us to conclude the ability of wild type and mutant proteins to interact. This question was addressed using a His60 Ni column pull-down assay. The panx1a-dTomato-His protein pulled down panx1a-EYFP efficiently (Figure 5C). The Y205A-dTomato-His protein showed a faint signal corresponding to panx1a-EYFP, suggesting that the ability to interact was drastically reduced. The negative control, dTomato-His paired with panx1a-EYFP protein lysates, did not pull-down wild type panx1a subunits excluding dTomato-His as interaction partner with either panx1a or EYFP.

## 4. Discussion

### 4.1. Panx1a Requires Aromatic Amino Acid Residues for Folding and Stabilization

The highly conserved aromatic amino acid residues located on the intracellular side of transmembrane domain 2 (TM2) and 3 (TM3) were identified by sequence alignment with human pannexin proteins and the INX-6 channel. Presently, only the high-resolution crystal structure of INX6 has been reported. The mutation of either the Y205 or the W123 residues resulted in the retention of the protein. Since the two residues are in close proximity to each other, as seen with the INX-6 channel, it is possible that they interact by aromatic–aromatic interactions similar to those reported previously [20]. We suspect that this interaction is needed for protein structure by enhancing the helix–helix association in a membrane environment. To prove this, we created mutants in which the aromatic residue was moved one or two residues upstream of the 205 position. Our data show that the position of the tyrosine residue is critical. When Y205 was mutated to another aromatic residue, phenylalanine, membrane localization was restored.

Many integral membrane proteins contain transmembrane domains made up of bundles of lipid-bilayer-spanning alpha helices. The TM alpha-helices are generally composed of a core of mostly hydrophobic amino acids, with basic and aromatic amino acids at each end of the helix. This arrangement is suggested to play a unique role in membrane protein interactions with water/lipid interfaces, anchoring the proteins into the membrane through an interaction of their aromatic rings with the lipid head groups [28,29,30]. Previously, aromatic–aromatic interactions have been shown to stabilize the transmembrane domains of the K^+^ channel [19]. The study explores the implications of these interactions for both channel function and stabilization. Similarly, a pore-blocking hydrophobic motif at the cytoplasmic aperture of the closed-state Nav1.7 channel was found [31]. However, a scanning cysteine-alanine mutagenesis (SCAM) analysis of the mouse Panx1 protein expressed in the *Xenopus laevis* oocyte model [32] has shown that replacing W127 and F220 with the amino acid cysteine had only minor effects on irreversible current inhibition with no loss-of-channel function after treatment with 100µM maleimidobutyryl-biocytin (MBB).

### 4.2. Lack of the Aromatic Amino Acid Residues Disrupts Trafficking and Limits Post-Translational Processing of Panx1a

Here, we present results that provide insight into the post-translational processing of panx1a and the relevance for trafficking. The presence of the high mannose glycosylation form (Gly1) on Western blots indicated the ability to undergo limited post-translational modification. Both W123A and Y205A mutant proteins still underwent the first modification step towards complex glycosylation (Gly2). N-glycosylation of rodent or human pannexin-1 at amino acid position N254 and the corresponding position N246 in zebrafish panx1a is considered necessary for trafficking to the cell membrane [9,10,14]. When lacking this N-glycosylation motif, both Gly1 and Gly2 forms were missing, and cell surface transport was limited [10,11,14,33].

The co-localization analysis revealed that the Y205A mutant was more abundant in the ER than the Golgi. Previous research has shown that the transport of Panx1 between the ER and Golgi is mediated via COP II vesicles [34]. When we tested the co-localization of Y205A with Sec24D, a COP II protein, a strong co-localization was found. Panx1 has also previously been shown to co-localize with the scaffolding protein caveolin-1 [35], which is the main component of the caveolae plasma membranes found in many cell types. Here, only the wild type panx1a protein co-localized efficiently, with significantly less co-localization of the Y205A mutant. The reduced co-localization with Cav-1 and lack of complex glycosylation implied that the Y205A mutant was not trafficking efficiently past the Golgi stage. This assumption was tested by blocking trafficking between ER and Golgi with Brefeldin-A treatment. Similar to previous reports [9], the spatial differences of intracellular Panx1 trafficking correlated with Golgi blockage by Brefeldin-A treatment.

### 4.3. The Y205A Trafficking Deficiency Was Not Caused by ER Stress

The accumulation of integral membrane proteins like panx1a can occur upon transient overexpression, causing ER stress when misfolded or when otherwise altered proteins trigger a cellular stress response. The RT qPCR analysis tested whether ER stress was a confounding factor in this investigation. Transcriptional regulation of Atf-4, CHOP, EDEM, BiP, and XBP1 is known as an indicator of ER stress and the unfolded protein response (UPR) [36]. Brefeldin-A treatment reliably upregulated the Atf-4, CHOP, EDEM, and BiP transcripts in Neuro 2a cells. In the case of XBP1, we also used primers for the spliced, unspliced, and total XBP1 mRNA [36]. When the XBP1 mRNA is spliced, it produces a transcriptionally active basic leucine zipper transcription factor that participates in regulating other ER stress genes. The spliced XBP1 was significantly upregulated after BFA treatment, validating our model. Both overexpression of the Y205A mutant or the wild type panx1a failed to cause transcriptional activation of ER stress genes. We excluded ER stress response as a primary cause of trafficking deficiencies of the Y205A mutant. Other types of protective or destructive stress responses, such as apoptosis, necrosis, pyroptosis, or autophagic cell death, were not investigated since transfected cells appeared healthy when the experimental endpoints were reached.

### 4.4. The Y205A Mutation Disrupted the Oligomerization State of the Panx1a Channel Assembly

Förster resonance energy transfer (FRET) tests and pull-down experiments tested the interaction between Y205A-EYFP and WT panx1a-DsRed protein pairs. In contrast to wild type:wild type pairs, the low FRET efficiency detected between wild type:Y205A, as well as Y205A:Y205A pairs, suggested that the mutant protein failed to form higher-order complexes. Consistently, the pull-down experiment demonstrated that the interaction between wild type and mutant proteins was low or even nonexistent.

## 5. Conclusions

This study identified two critical aromatic amino acids, W123 and Y205, that together help panx1a proteins to oligomerize and efficiently traffic to the cell membrane. Based on the high-resolution structure of the INX-6 channel [26], it is reasonable to speculate that the aromatic–aromatic interaction between Y205 and W123 contributes to panx1a channel folding or assembly. These findings provide new insights into the life cycle of pannexins by complementing a growing body of distinct molecular mechanisms and requirements, enabling regulation of Panx1 oligomerization, glycosylation, and trafficking.

## Figures and Tables

**Table 1:** List of primers for *panx1a* mutagenesis.

Gene	Primer ID	Primers *
*panx1a*	Y205A_Fwd	ACTGATCCGCgccCTTCTTTGCC
	Y205A_Rev	AATCCTTTAGAGTAGCGC
	R204Y:Y205R_Fwd	ATTACTGATCtaccgcCTTCTTTGCCGCAC
	R204Y:Y205R _Rev	CCTTTAGAGTAGCGCTTG
	I203Y:R204I:Y205R _Fwd	ccgcCTTCTTTGCCGCACCATC
	I203Y:R204I:Y205R _Rev	atgtaCAGTAATCCTTTAGAGTAGCG
	W123A_Fwd	AGCGTTGTTTgcgCGGTTTACAG
	W123A_Rev	GGCATGTAAACTGACACTG
	Y205F_Fwd	ACTGATCCGCttcCTTCTTTGCC
	Y205F_Rev	AATCCTTTAGAGTAGCGCTTG

* nucleotides in small letters indicate the changes introduced into the wild type (WT) sequence.

**Table 2:** List of primers used by real-time qPCR to detect endoplasmic reticulum (ER) stress markers.

Primer ID	Primers
ATF4_Fwd	GGGTTCTGTCTTCCACTCCA
ATF4_Rev	AAGCAGCAGAGTCAGGCTTTC
CHOP_Fwd	CCACCACACCTGAAAGCAGAA
CHOP_Rev	AGGTGAAAGGCAGGGACTCA
EDEM_Fwd	CTACCTGCGAAGAGGCCG
EDEM_Rev	GTTCATGAGCTGCCCACTGA
BiP_Fwd	TTCAGCCAATTATCAGCAAACTCT
BiP_Rev	TTTTCTGATGTATCCTCTTCACCAGT
sXBP1_Fwd	CTGAGTCCGAATCAGGTGCAG
sXBP1_Rev	GTCCATGGGAAGATGTTCTGG
usXBP1_Fwd	CAGCACTCAGACTATGTGCA
usXBP1_Rev	GTCCATGGGAAGATGTTCTGG
tXBP1_Fwd	TGGCCGGGTCTGCTGAGTCCG
tXBP1_Rev	GTCCATGGGAAGATGTTCTGG

## Supplementary Materials

The following are available online at https://www.mdpi.com/2218-273X/10/2/272/s1, Figure S1: Topological comparison of panx1a with its ohnologue panx1b, human pannexin orthologs, and the *C. elegans* INX-6 protein. Figure S2: FRET distance graph shows the FRET distance using the reference distance between DsRed and EYFP pair (4.9nm).
