# Supplementary material for: Role of an Aromatic–Aromatic Interaction in the Assembly and Trafficking of the Zebrafish Panx1a Membrane Channel
Source: Biomolecules. 2020 Feb 11;10(2):272. doi: 10.3390/biom10020272 (PMC7072349; doi:10.3390/biom10020272)
Supplement: Supplementary file 1 [file biomolecules-10-00272-s001.pdf]

biomolecules

Supplementary Files

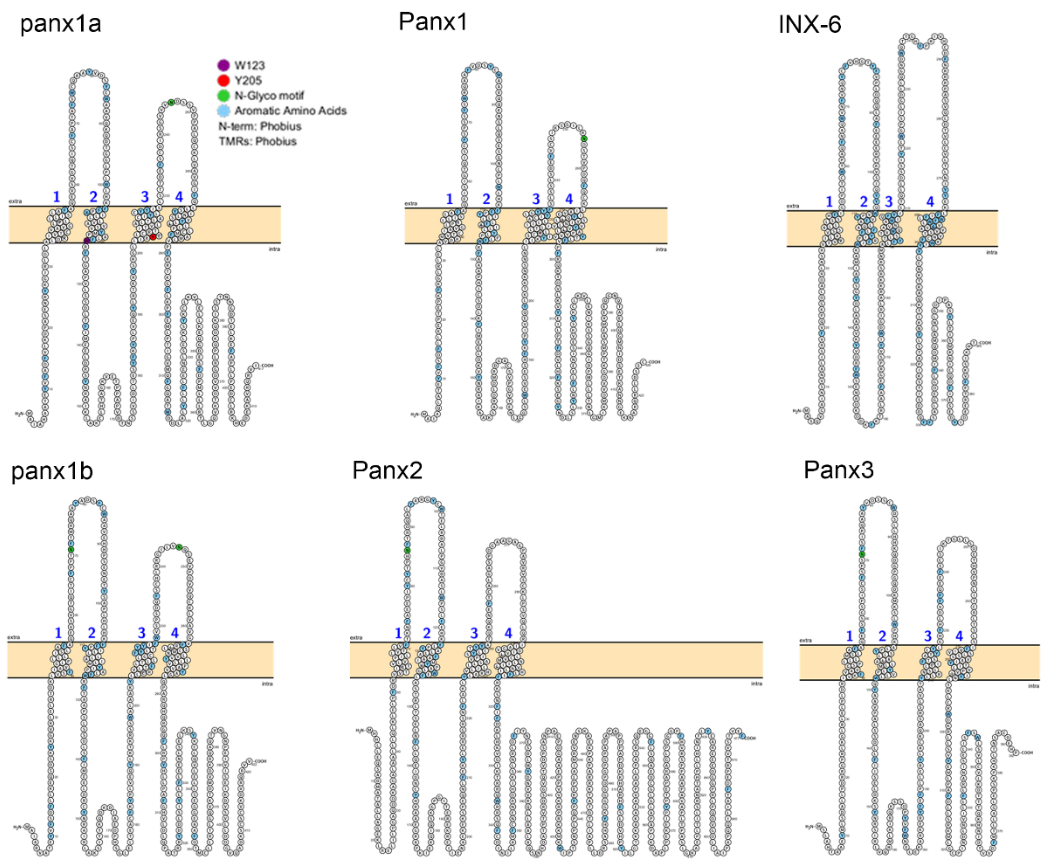

Supplementary Figure 1. Topological comparison of panx1a with its ohnologue panx1b, human pannexin orthologs, and the *C. elegans* INX-6 protein. The aromatic residues W123 and Y205 are highlighted in panx1a in purple and red, respectively. Aromatic residues (tyrosine, tryptophan, and phenylalanine) are highlighted in blue. Images were generated using the interactive Protter tool [27].

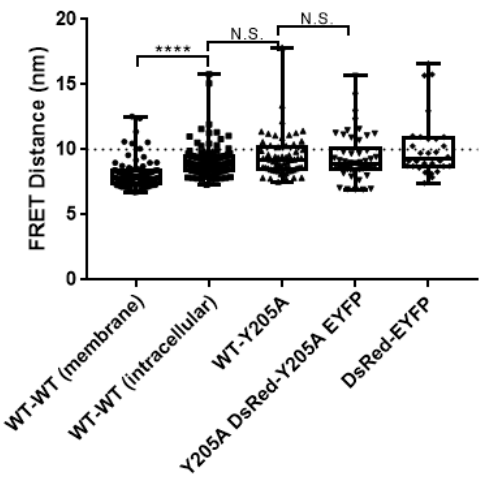

Supplementary Figure 2. FRET distance graph shows the FRET distance using the reference distance between DsRed and EYFP pair (4.9nm). WT-WT (membrane) indicated panx1a-DsRed interaction with panx1a-EYFP using selected cell membrane regions as a positive control. WT-WT (intracellular) indicated panx1a-DsRed interaction with panx1a-EYFP using selected regions inside the cell. WT-Y205A indicated panx1a-DsRed interaction with Y205A-EYFP using selected regions inside the cell. Y205A-Y205A indicated Y205A-DsRed interaction with Y205A-EYFP inside the cell. Note that only WT:WT pairs in the cell membrane showed proximity indicating a compact assembly of panx1a monomers in channels. DsRed-EYFP pairs serve as negative control. The dotted line represents the FRET distance threshold of 10nm. ****$p<0.0001$, N.S., not significant.
